# Could a culturally and age-appropriate song contribute towards malaria prevention in primary school learners?

**DOI:** 10.1186/s12936-022-04120-x

**Published:** 2022-04-02

**Authors:** Chad M. Anderson, Irma Eloff, Taneshka Kruger

**Affiliations:** 1 Faculty of Health Sciences, School of Health Systems and Public Health, University of Pretoria Institute for Sustainable Malaria Control, University of Pretoria, Pretoria, South Africa; 2 Department of Education Psychology, Faculty of Education, University of Pretoria, Pretoria, South Africa

**Keywords:** Malaria prevention, Malaria education, Music education, Disease prevention, Knowledge retention, Behavioural change, Interventions, Questionnaires

## Abstract

**Background:**

Although it is a preventable disease, malaria continues to present one of the major global health challenges. The disease is especially deadly to children under the age of 5 years. This is partly due to the fact that they have yet to build up an immune system to help protect them against malaria. As a prevention strategy, music is one way of communicating knowledge to young children and could therefore potentially be used to change children’s behaviour in order to avoid getting sick from malaria. This exploratory study aimed to compare intervention strategies designed to educate and improve knowledge growth about malaria and its prevention in Grade 3 learners in a high-risk malaria region in Vhembe District, Limpopo Province, South Africa.

**Methods:**

Various intervention strategies were employed and evaluated to determine the most effective intervention method. The study was split into two Phases. Phase 1 used a culturally and age-appropriate song as an intervention, along with a drama piece, a song and drama piece combination, and a control group. The most effective intervention strategy determined during Phase 1 was then subjected to Phase 2, against a control group to validate its efficacy. Questionnaires were used during pre-intervention and post-intervention interviews to evaluate the knowledge growth, if any, of the learners of selected primary schools in two different areas in Vhembe District. Pre-intervention interviews were followed by a specific intervention, based on the respective study groups. After 6 weeks, the post-intervention interviews were done to determine how much learners managed to learn and retain from the intervention received.

**Results:**

The study found that the group that had only the song as intervention was the most effective learning intervention method in both Phases 1 and 2. Both phases showed that there was statistical significance in almost all of the nine questions asked on the questionnaires.

**Conclusions:**

The study concluded that a culturally and age-appropriate song can play a significant role in developing behavioural changes and spreading awareness against disease in a high-risk malaria region.

## Background

Malaria, a global public health concern, is caused by the bite of a female infected *Anopheles* mosquito. The disease is treatable and can be prevented using local community health strategies of clearing bushes and draining stagnant water near residents. According to the World Health Organization (WHO), more than 229 million malaria cases were reported globally, with more than 409,000 deaths in 2019 [1]. According to WHO, malaria cases have increased over the past years despite efforts to eliminate the disease due to drug resistance, insecticide resistance and challenges in malaria funding [2]. Children below the age of 5 years are highly susceptible to contracting malaria, which accounted for 67% of malaria fatalities (409,000 deaths) worldwide, approximately 274,030 deaths in 2019 [3].

Recent research has sought to help people with diseases such as malaria to live more fulfilling lives. Research about malaria has found that singing or listening to a song provides behavioural and emotional benefits to people with different diseases. Music has the potential to reduce agitation, anxiety, and depression and can also relieve stress [4]. Simple songs have been used to educate people on how to fight and eradicate malaria across Africa. In Mali, music is used as a “way of life”. People, who compose music, including West Africa traditional storytellers and pop stars, are used as change agents in disseminating information about an evidence-based project or behaviour [5].

Many communities have composed and sung songs to change attitude, behaviour, to control and eliminate malaria. People in Uganda, specifically Amolatar District, in Northern Uganda, treat people by dance performance of a lively song to combat and prevent malaria. In 2014, Anthony Okello joined the Uganda Initiative programme of malaria supported by the US President’s Malaria Initiative (PMI) in Uganda [6]. He composed a song and lyrics to communicate the importance of spraying insecticide to eradicate the mosquito, which transmits the deadly disease. In Tanzania, many artists came together and composed a song with a message of preventing and stopping the spread of malaria [6]. Their song was named *Malaria Haikubaliki*, which is a Swahili phrase meaning malaria is unacceptable. They advocated for the government to take responsibility for leading the nation in the fight against the disease to make the country mosquito-free.

Composing an age- and culturally appropriate song has thus been thought to assist in sending a message to children from the age 5 and above [7]. In doing so, malaria and early childhood education experts and community representatives select messages relevant to children, which is a determinant for changing children’s behaviour. Appropriate measures are established to ascertain the effectiveness of the recommended song [8]. There have been other songs about malaria and other diseases, but they were not always specific or age-appropriate, as was done in this study. The songs were found to not have been developed through scientific research methods, but rather for the purpose of advocacy. Thus, the song in this study was the first song composed for malaria awareness (and potential prevention) through a scientific method, targeting a specific age group [8].

In South Africa, malaria is not a dangerous threat, but preventive measures should be taken. The government intends to reach its elimination goal despite current staffing shortages and supply chain challenges. Through the regional Elimination 8 (E8) initiative, 14 border posts have been set up in high-risk areas to improve access to timely malaria diagnosis and treatment for mobile and migrant populations, reducing the risk of onward transmission [9]. The Limpopo Province was purposefully selected for this study due to the site area having the highest malaria incidence and prevalence in South Africa. The Limpopo Province reportedly was at a historical risk, however, indoor residual spraying (IRS) has restricted the disease to the eastern and northern areas of Mopani and Vhembe districts of the Province [10].

A research project conducted in Malawi studying the socio-economic impacts of malaria revealed that the level of education and household wealth index had an impact on a population’s malaria morbidity index. People living in South Africa’s rural malaria-endemic areas share similar socio-economic characteristics as people in Malawi, therefore, the same can be said about educating people in South Africa about malaria. Results from the study showed that when a community is educated about malaria, the risk for contracting malaria decreases. There is a need for malaria prevention education for those people at risk of malaria. The study suggested when pregnant mothers are educated regarding prenatal care, information on childcare should include malaria prevention [11]. Since mother and child are both at risk of having malaria, education is essential as early as possible.

Since, currently, there is no vaccination to prevent malaria, proactive prevention of malaria is considered an action-oriented solution. Despite proactive prevention, many individuals still get infected with malaria and die. Research indicates, one of the preventive measures that WHO recommends is wearing long-sleeved clothing and socks when outside at night [12], thereby reducing exposure of the skin from which the mosquito can draw blood. WHO findings indicate that mosquito nets are an effective way of thwarting mosquitoes and keeping them away from the skin. If nets are not available, WHO recommends covering the skin with light clothing as the most sustainably preventative measure, and especially so for individuals that sleep outside during the hot summer months [2].

In this exploratory mixed-methods study, the research first aimed to compare and evaluate three teaching intervention methods to assist in educating young children, specifically Grade 3 (usually 10–11 years of age) primary school learners, about malaria. The study sought to assess one teaching intervention in terms of its efficacy to teach young children how malaria is spread and about the ways to prevent being infected with malaria, to potentially enable behavioural changes in themselves and their communities.

## Methods

### Study design

#### Selection of study area

**Fig. 1:**
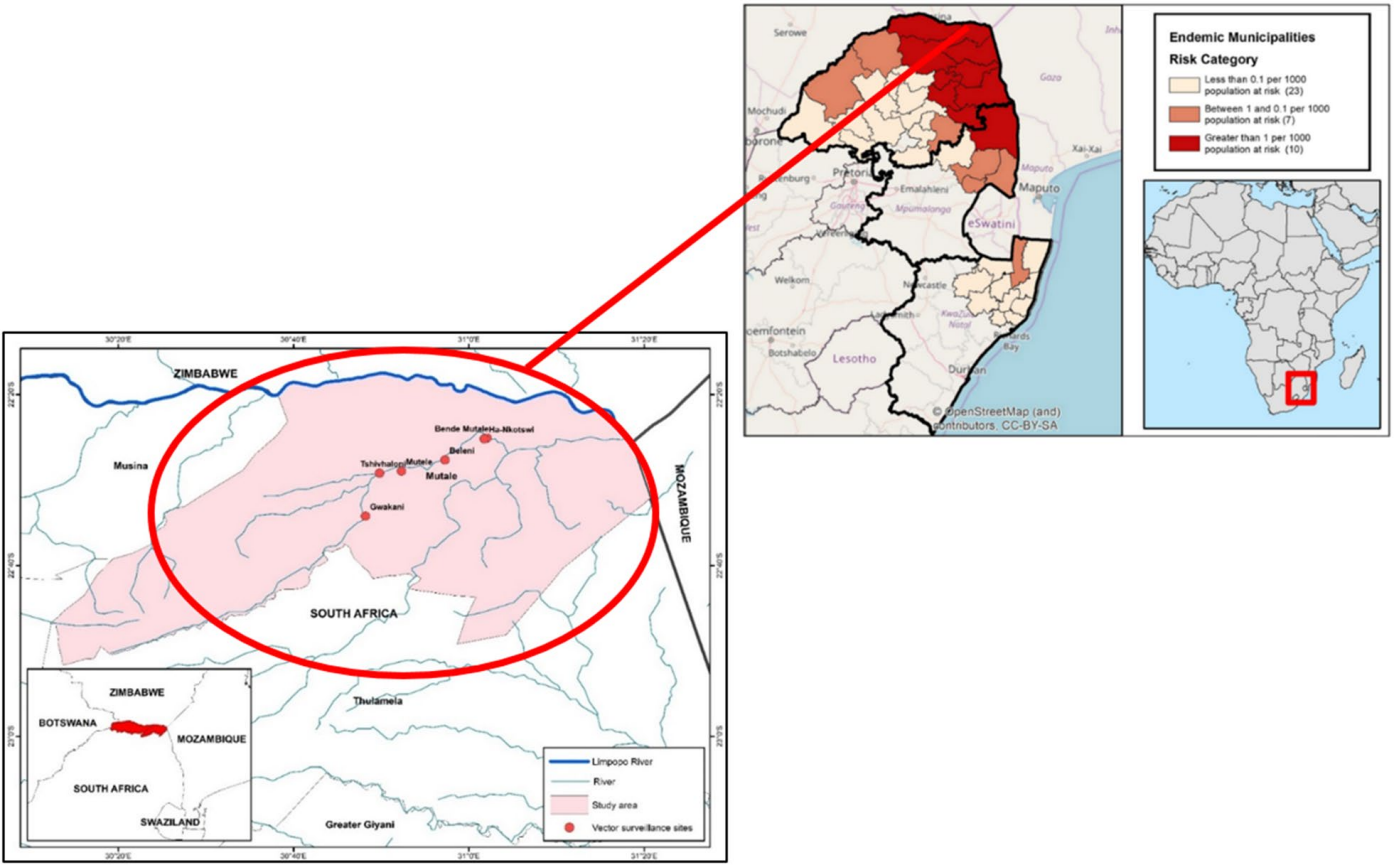
Map of the Vhembe District in Limpopo, South Africa

The study area selected in both Phases 1 and 2 was in the Limpopo River Valley (LRV) region in Vhembe District, Limpopo Province, on opposite sides of the Soutpansberg Mountain, South Africa. Phase 1 was conducted in the Niani Circuit on the north side and Phase 2 in Sambandou Circuit on the south side of the mountain. The LRV region in Vhembe District was purposely selected due to it having the highest malaria incidence and prevalence in South Africa [13]. The Vhembe District can be seen in Fig. 1.

#### Identification and approval of target schools

The Limpopo Department of Basic Education Circuit Managers for the Niani and Sambandou Circuits identified schools in the areas to be a part of the study. After obtaining approval by the respective authorities, the schools were mapped out using GPS to establish which were closest together for each intervention group. The schools were then randomly assigned a study group based on their locations, with the control groups being the farthest away from all other schools.

#### Flow of the study

The study was divided into two phases. Phase 1 was to decide which intervention method out of three plus a control was the most effective to teach young learners about malaria. Phase 1 involved a pre-intervention questionnaire-based interview of all participants. Questions for pre- and post-interventions were exactly the same (Table 1). The questionnaire was administered by local Vhavenda women field assistants who had been trained in data collection through the use of questionnaires. This pre-intervention interview was the baseline assessment of what participants knew about malaria at the start of the study. The participants were Grade 3 learners from different primary schools. The Grade 3 teachers were also interviewed to determine their level of knowledge of malaria and prevention strategies. They were directed to determine how their administration of the intervention could impact on learners’ ability to gain knowledge from the intervention.

**Table 1:** Questions in pre-and post-intervention questionnaires during Phases 1 and 2

Question number	
1	Have you ever heard about malaria?
2	What do you know about malaria?
3	Which insect causes malaria?
4	The insect causes malaria by?
5	What does malaria feel like (sickness)?
6	Where do mosquitoes come from?
7	What time do mosquitoes come outside?
8	What are a few ways to stop malaria?
9.1	I know how I can protect myself from malaria?
9.2	Why? Why not?

After the pre-intervention interview, three types of interventions (song, drama piece, song-and-drama piece) were provided to some of the schools, based on the study group assigned to that school. An existing song that was developed through Participatory Action Research (PAR) was used in this research [8]. PAR ensured that the song was both culturally and age-appropriate for the learners by using different experts in malaria (public health/disease experts), education (early childhood education), social anthropology, and music, to review the song until the content and format was agreed upon. Six weeks after the interventions, a post-intervention questionnaire was given to all the learners to determine if any knowledge was gained on malaria with their given intervention and how much was retained, if any, using the exact same questionnaire as used in the pre-intervention interview. The same process was followed for the post-intervention interview. Data from Phase 1 were then analysed and used to develop a research plan for Phase 2 of the study.

**Fig. 2:**
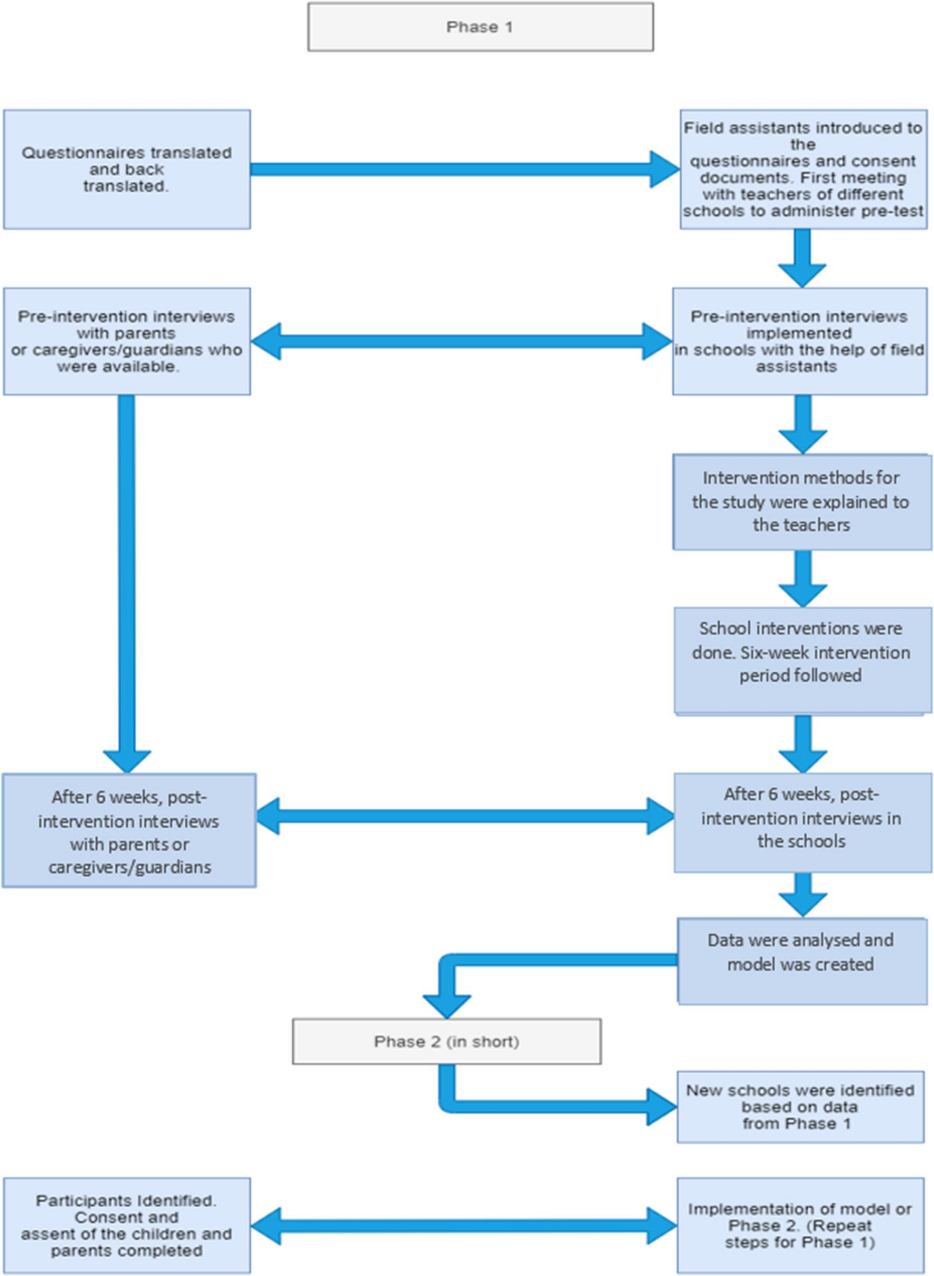
Flow diagram model summarizing Phases 1 and 2 of the research study

Once Phase 1 was completed, the most effective intervention method of the three interventions was identified. Phase 2 was done for the validation of the findings of Phase 1, to conclude the most effective learning method to teach young learners about malaria. Phase 2 followed Phase 1 having pre-intervention interview with a 6-week intervention (only the most effective one identified from Phase 1) followed by the post-intervention interview to determine if participants had gained any knowledge from their given intervention and how much retention had occurred using the same questionnaire. A flow diagram summarizing Phases 1 and 2 is shown in Fig. 2.

## Phase 1

### Selection of the target schools

Phase 1 of the study was carried out in the rural Vhembe District, Limpopo Province, north of the Soutpansberg. Grade 3 learners from 12 primary schools were selected for this respective phase asked the pre- and post-intervention questionnaire to determine their malaria knowledge. The primary schools selected for Niani Circuit included: Bale, Gumbo, Madavhila, Madimbo, Malale Manenzhe, Masea, Masisi, Matshena, Mutele B, Sigonde, and Tshiungani.

### Breakdown of interventions

The 12 selected primary schools were divided into four study groups with three schools per group. Three of these groups received interventions after the pre-intervention interview was administered, whilst the fourth group was a control group, receiving no intervention.

### Data collection

**Fig. 3:**
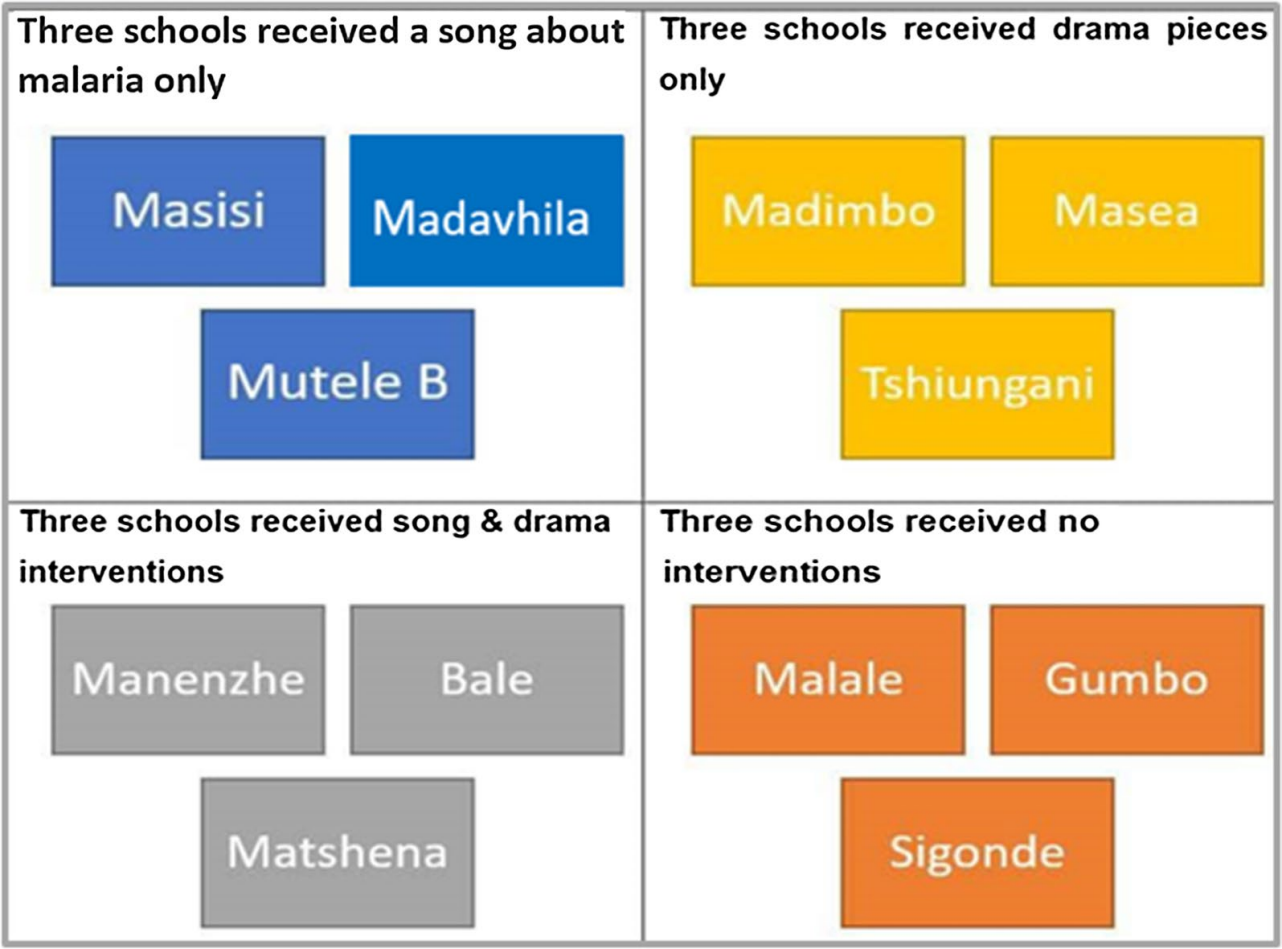
Breakdown of intervention methods for the 12 primary schools enrolled during Phase 1 of the study

Data were collected from children of the 12 selected primary schools through pre- and post-intervention interviews. After pre-intervention interviews, the Grade 3 teachers implemented the intervention (song, drama piece or song-and-drama piece) in each of the classrooms, with the researcher and the field assistants, in the morning before classes began, at a time decided upon in order to avoid disrupting the scheduled school day. The teachers were reminded of the importance of malaria education to prevent disease transmission and their roles in implementing such interventions. The interventions were followed by a 6-week intervention period. There were three different types of interventions and a control group. The interventions were broken down into four school groups including the schools that received only the malaria song; schools that received only a drama piece; schools that received both song and drama piece; and, schools that received no intervention (control group) (Fig. 3). After the 6 weeks of the intervention, the post-intervention interviews were done and data were stored to be analysed.

The Grade 3 teachers were also asked the same pre-intervention questionnaire as the learners in order to determine their malaria knowledge baseline. However, the post-intervention questionnaire differed from that administered to the learners. This alternative questionnaire included questions about what the teachers noticed during the time prior to the post-intervention interviews. Moreover, in order to determine if children had shared their knowledge with members of their family rather than keeping it to themselves, some children’s parents/caregivers/guardians were also questioned. These participants were interviewed but not exposed to any of the interventions prior to the post-intervention questionnaire.

## Phase 2

### Selection of the target schools

Phase 2 of the study was done in the rural Vhembe District, Limpopo Province, south of the Soutpansberg. Grade 3 learners from eight different schools in the area were asked the pre- and post-intervention questionnaire to determine their malaria knowledge. The primary schools identified for Niani Circuit included: Karel Ngigideni, Khavhambe, Lamvi, Lavhurala, Mavunde, Tshikalange, Tshikondeni, and Vhuri-vhuri.

### Breakdown of interventions

From the results in Phase 1, the most effective intervention method was determined for knowledge gained and retention in order to effect behavioural change. Results indicated that the song used as an intervention on its own was the most statistically significant. Phase 1 was a proof-of-concept study. The results were used to establish how well the young children were able to learn from the song or other intervention based on the results obtained during the Phase 1 part of the study through analysis with a statistical regression modelling programme.

**Fig. 4:**
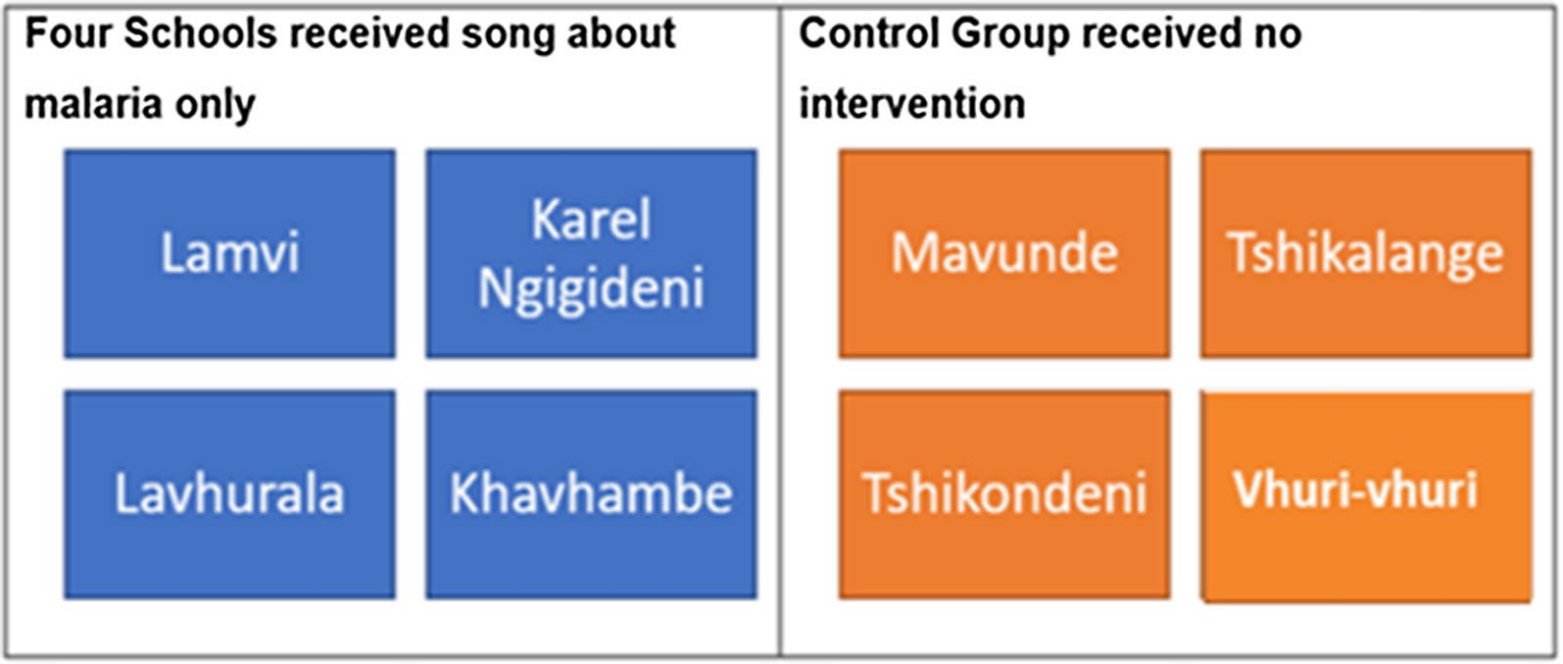
Breakdown of intervention methods for the eight primary schools enrolled during Phase 2 of the study

The eight selected primary schools were divided into two groups with four schools per group. One group received only the song and the other group was the control and received no intervention. The breakdown of the schools per intervention group during Phase 2 can be seen in Fig. 4.

The reason the control group was included in the post-intervention was to determine if any learner from the control schools had obtained new knowledge about malaria since the pre-intervention interview. If any learner showed knowledge gained, that should indicate that malaria knowledge was gained by another method, excluding the different interventions. This could include: ‘cross-contamination’ between participants from different schools where the interventions were implemented: someone they knew became sick with malaria after the pre-questionnaire was implemented, or they heard about malaria while at a clinic for other issues, or the study conducted in the region may have raised general awareness.

### Data collection

The data were collected from children of the eight selected primary schools through pre- and post-intervention interviews. The pre-intervention interview was followed by introducing the song to the intervention group. The control group received no intervention. A 6-week period elapsed before a post-intervention interview was conducted, as in Phase 1. The Grade 3 teachers implemented the intervention in each of the classrooms with the researcher and the field assistants as in Phase 1.

In addition to collecting data from the children, some parents/caregivers/guardians of the children were asked the questionnaires as in Phase 1. The purpose was, as for Phase 1, to determine if the children shared the knowledge outside the school environment (at home) or if the parent’s knowledge of malaria changed after the children were taught the interventions. This was, however, not the main aim of the study, but provided additional background information.

## Data and statistical analysis

The exploratory study employed a mixed methods methodology using both qualitative and quantitative data collection. The preferred class sample sizes for Phases 1 and 2 were determined statistically to be n = 20 and n = 16 learners per class, respectively. Based on the data collected from Phase 1, it was determined that 16 per class would be needed to complete Phase 2.

In a hierarchical two-level mixed effects model with four clusters in groups 1 and 2, respectively, and 16 subjects per cluster, 90.19% power is achieved to detect the difference in proportions of at least 0.4, where groups 1 and 2 proportions are 0.9 and 0.5, respectively, assuming that is 0.1 and that the regression model is performed at the 5% significance level.

Data analysis employed mixed effects regression to determine the relationship of text score with fixed effects teaching mode and observation timed.

The study had open-ended and close-ended responses due to it being a mixed method study. The closed-ended responses were entered on an Excel sheet and statistical analyses were done on the data in IBM SPSS 2. Participants were specified as random effects with an intercept and independent/unstructured covariance. The mixed end margins were employed, and any text done was at the 05 level of significance.

The open-ended responses were sorted based on their answers in Microsoft Excel. Once sorted in Microsoft Excel, the data were coded, and from there the newly coded data were quantitatively analysed. Selected quotes highlighting deep and meaningful expression of information noticed by the teacher intervention groups during their interventions, taken from the pre- and post-intervention interviews, were also used.

In order to assess if there was any improvements in the answers of learners between the post intervention, the McNemar test was applied, using the before and after intervention results as test variables [14]. For this test, the null hypothesis states that for each group (control, song, drama, song-and-drama) the pre- and post-intervention questionnaire are equally effective. A p < 0.05 would indicate that there were statistically significant differences between the pre- and post-intervention questionnaires and thus, the interventions were effective.

## Results

### Phase 1

The data obtained from the pre- and post-intervention questionnaire were subjected to analysis to determine the most effective learning method for the young children. A statistically significant difference between the pre- and post-intervention questionnaires was indicated by p < 0.05, thus demonstrating the interventions were effective.

**Fig. 5:**
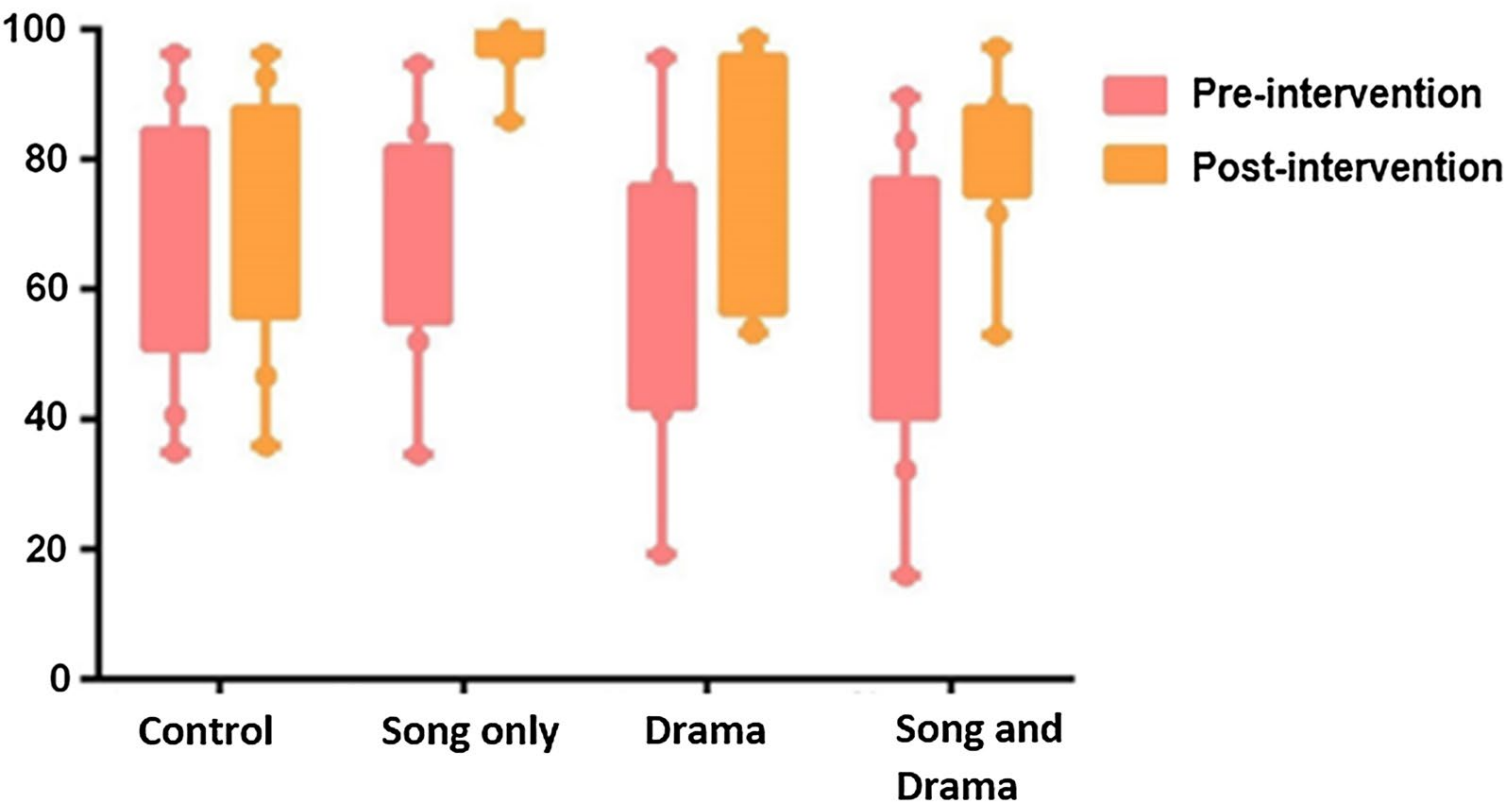
Correct answers shown as mean percentage of all questions from the questionnaire percentages compared by intervention groups from pre-intervention to post-intervention questionnaires for Phase 1

The pre-intervention questionnaires and post-intervention questionnaires had nine questions that remained the same for both pre- and post-intervention interviews. The results from each question based on the four intervention groups were obtained and compared. Amongst all the four interventions groups, only the group that received only the song, showed statistical significance (p < 0.05). Pre- and post-intervention comparison of the song only group is shown in the Table 2 where a score of p < 0.05 shows statistical significance. For the song only group, the learners’ answers improved significantly (p < 0.05) for all questions, except Question 3 (Which insect causes malaria?). However, the non-significance on this item is most likely attributed to the fact that even before the intervention, the percentage of correct answers was high (93%). As compared to the control, all the groups had an increase in the percentage of correct answers. The song only group had the most and highest improvements for correct answers (Fig. 5).

**Table 2:** Pre- and post-intervention comparison of the group that received the song-only intervention in Phase 1 (n = 244)

Questions	Correct answers before the intervention (%)	Correct answers after the intervention (%)	Answer improvement (%)	p value*
1: Have you ever heard about malaria?	54	100	46	< 0.001*
2: What do you know about malaria?	70	100	30	0.002*
3: Which insect causes malaria?	93	100	7	0.125
4: The insect causes malaria by:	80	100	20	< 0.001*
5: What does malaria feel like (sickness)?	52	97	45	< 0.001*
6: Where do mosquitoes come from?	72	97	25	< 0.001*
7: What time do mosquitoes come outside?	87	98	11	0.039*
8: What are a few ways to stop malaria?	34	87	53	< 0.001*
9.1: I know how I can protect myself from malaria?	75	100	25	< 0.001*
9.2: Why? Why not?	74	98	24	0.001*

*Statistically significant p values, p < 0.05

### Phase 2

The result for Phase 2 were measured as in Phase 1. The McNemar test was applied once again, using the pre-and post-intervention questionnaire results as test variables [14]. For this test, the null hypothesis states that for each group (control and song only), the pre- and post-intervention questionnaires are equally effective. A p < 0.05 would show that there was a statistically significant difference between the pre-and post-intervention questionnaire and thus, the interventions were effective. The questions on the pre- and post-intervention questionnaires remained the exact same as Phase 1.

The control group had two questions with a statistical significance: in Question 1 (Have you ever heard about malaria?), and Question 9.1. (I know how I can protect myself from malaria?). These questions were yes or no answers. Question 2 (What do you know about malaria?) and 9.2 (Why? Why not?) were to ensure that Questions 1 and 9.2 had accurate information. Therefore, with Question 9.2, the learner’s knowledge decreased for the Why section from Question 9. The children could not accurately demonstrate that they knew the prevention methods. Question 2 saw some of the children give more correct answers with a slight increase of 8% but it did not show a statistical significance. The remaining questions had slightly higher percentages of correct answers, but not higher than an 11% increase and no statistical significances (Table 3).

**Table 3:** Pre- and post-intervention comparison of the control group in Phase 2 of the study (received no intervention)

Question number	Correct answers before the intervention (%)	Correct answers after the intervention (%)	Answer improvement (%)	p value*
1: Have you ever heard about malaria?	52	84	32	< 0.001*
2: What do you know about malaria?	76	84	8	0.754
3: Which insect causes malaria?	89	100	11	0.016
4: The insect causes malaria by:	80	80	0	N/A
5: What does malaria feel like (sickness)?	54	59	5	0.607
6: Where do mosquitoes come from?	69	80	11	0.167
7: What time do mosquitoes come outside?	69	69	0	N/A
8: What are a few ways to stop malaria?	28	30	2	1.000
9.1: I know how I can protect myself from malaria?	74	97	23	0.001*
9.2: Why? Why not?	87	78	− 9	0.791

*Statistically significant p values, p < 0.05

For the group that received the song only as an intervention, all the questions except Question 2, 3 and 9.2 had a statistical significance in knowledge retention. However, Questions 3 and 9.2 already had a higher percentage in their pre-intervention questionnaire and their answers still increased. Question 2 had a 20% increase and saw numerous learners demonstrate they learned about malaria, but it was not high enough to show statistical significance (Table 4).

**Table 4:** Pre- and post-intervention comparison of the song-only group, i.e., song-only intervention in phase 2 of the study (n = 124)

Question number	Correct answers before the intervention (%)	Correct answers after the intervention (%)	Answer improvement (%)	p value*
1: Have you ever heard about malaria?	41	100	59	< 0.001*
2: What do you know about malaria?	77	97	20	0.125
3: Which insect causes malaria?	92	100	8	0.063
4: The insect causes malaria by:	75	100	25	< 0.001*
5: What does malaria feel like (sickness)?	43	98	55	< 0.001*
6: Where do mosquitoes come from?	63	100	37	< 0.001*
7: What time do mosquitoes come outside?	75	100	25	< 0.001*
8: What are a few ways to stop malaria?	10	87	77	< 0.001*
9.1: I know how I can protect myself from malaria?	57	100	43	< 0.001*
9.2: Why? Why not?	92	98	6	0.250

*Statistically significant p values, p < 0.05

**Fig. 6:**
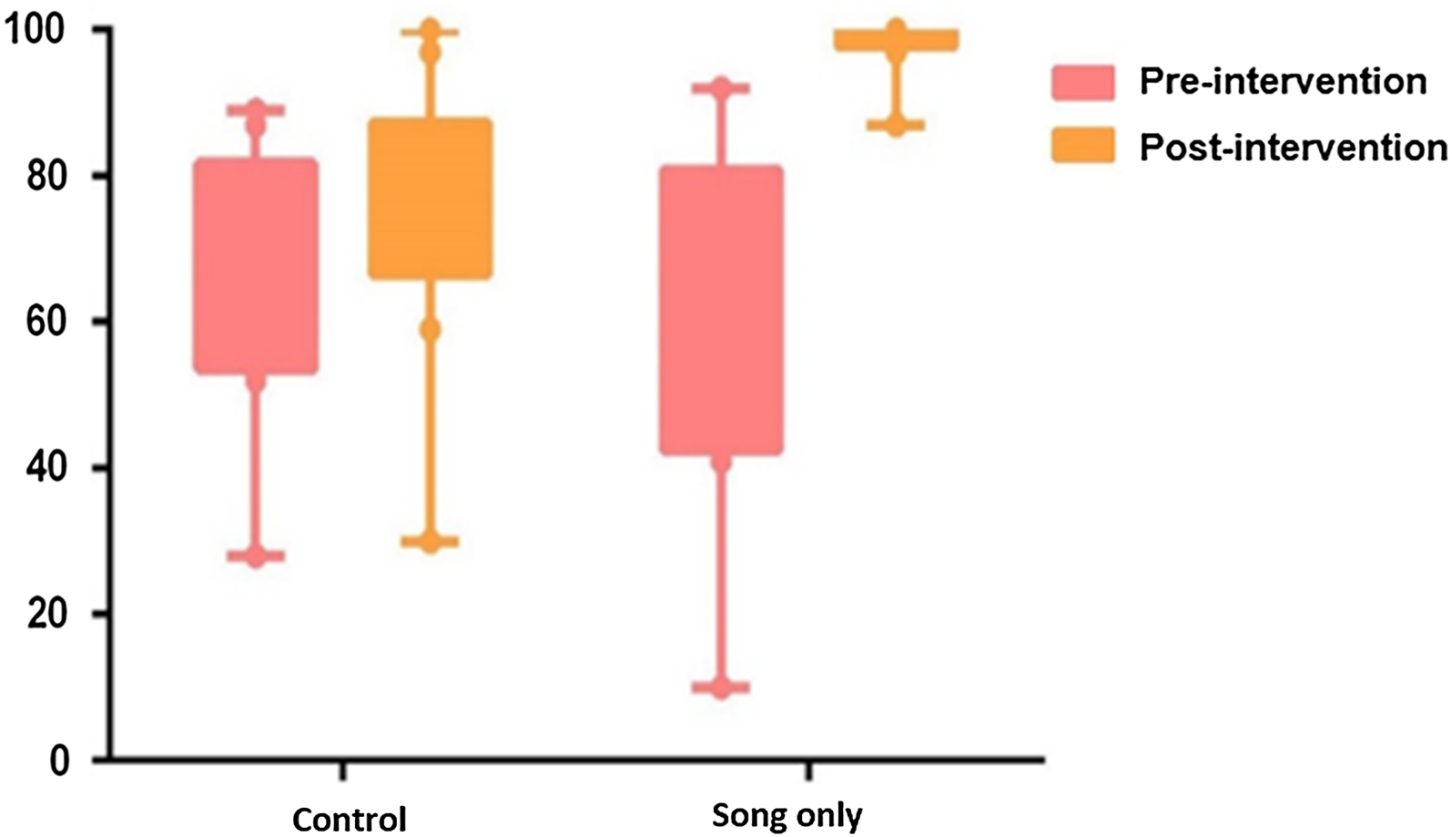
Correct answers shown as the mean of all questions from the questionnaire percentages compared by intervention groups from pre-intervention to post-intervention questionnaires for Phase 2

Comparing the control to the song only group, the song only group had the highest increase in the percentage of correct answers for all nine questions. The song only pre-intervention questionnaire had several wrong mean answers, and the post-intervention questionnaire had all high correct mean answers compared to the slight increase of correct answers in the control group (Fig. 6).

The research study also engaged teachers and parent/guardians/caretakers as participants to support or focus the young learners. A teacher’s knowledge has a critical impact on informing children about malaria prevention. The research study revealed that the intervention helped teachers to learn too and to teach in the most creative way. The findings revealed the positive impact of children’s behavioural change in creating a ‘ripple effect’ as results obtained by the interviews of the parents to look into potential passing on of information from learners to other family members indicated increase in knowledge gain. This is significant as the parents’ group for the song scored a statistical significance. The parents mentioned that they heard the song at home and demonstrated knowledge growth based on just hearing the children singing at home.

## Discussion

**Fig. 7:**
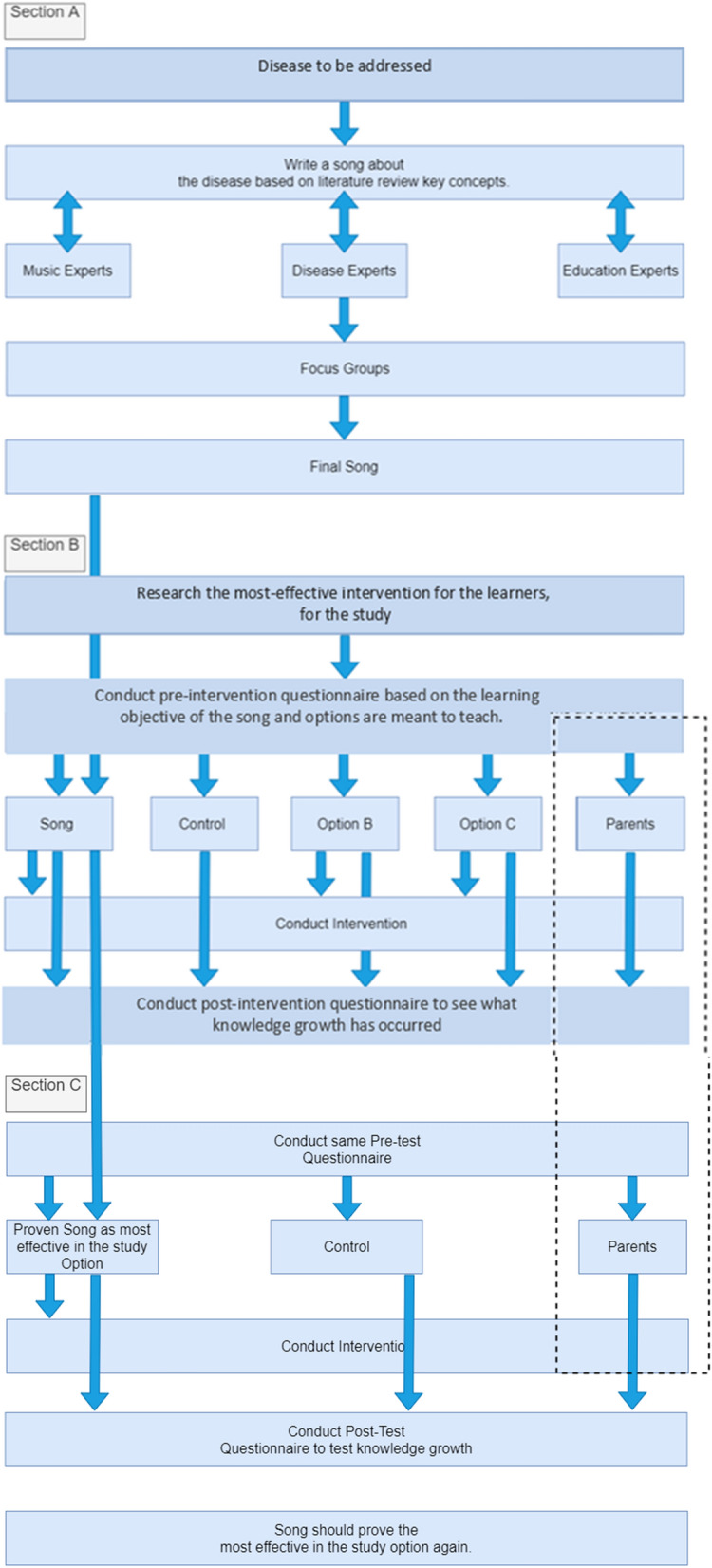
Section A, B and C Flow chart of the entire study including the steps on developing the culturally and age-appropriate song, from the development of song to the end of Phase 2

This study aimed to determine the possibility of a culturally and age-appropriate song being able to convey important messages to vulnerable communities, in this case young children, on how to prevent malaria transmission. The song used was developed in a previous study through the PAR approach [8]. This approach ensured that the song was both culturally and age-appropriate for the learners by using different experts in malaria, education, social anthropology, and music to review the song until the content was agreed upon. This specific song was deemed age-appropriate for children above the age of 5 years, cognitively from ages 8 to 13. Different age groups would require different songs. For example, children under 5 would require a more simplified and shortened version of the song used in this research [8]. The song was used in real-life settings in primary schools in a high-risk malaria area to determine if there is any knowledge growth in what learners knew about malaria and ways to prevent getting the disease. The entire process is summarised in Fig. 7. The Figure illustrates the process from step 1, to develop the culturally and age-appropriate song for a specific target audience, as described by Anderson et al. [8]. The process continues showing how the song was trialled in schools to determine its effectiveness in teaching about a disease, in this case malaria, as mentioned previously songs have been used before in disease prevention. However, songs about malaria are not always target-specific or age-appropriate, indicating they have not been developed through research method. The song used in Phases 1 and 2 of this study was developed through the use of participatory action research to ensure it was age- and culturally appropriate. The same song was used in the study areas and measured how significant it was in knowledge growth from questionnaires with malaria-related questions.

The results of Phase 2 indicated that all the learners in the song only group were able to learn about malaria and had substantial knowledge growth in some questions during their pre-intervention to post-intervention questionnaires. For example, in Question 8, 10% of children in the song only group knew all the correct symptoms of malaria. The control group managed to score a higher percentage in the pre-intervention with 28% of the children correctly knowing the prevention methods. Therefore, more children in the control group started off knowing more about the prevention methods compared to the song only group.

Upon follow-up in the post-intervention questionnaire for the song only group, it was concluded that 87% of the children demonstrated that they had learned all the correct prevention methods. This would be a 77% increase from the pre-intervention questionnaire in correct answers for the song only intervention. The largest percentage increase in the study. This was compared to 30% of children knowing the correct prevention methods, a 2% increase of knowledge growth in the control group. However, in the control group, the smallest increase in knowledge growth could never reach more than 11% for a particular question. The song only intervention reached 100% in knowledge growth of the children in six questions for the post-intervention questionnaire and in another three questions, only one or two children missed the concept correctly, thus, further validating the results that the song only intervention helps with memory retention and knowledge growth of the children. The song only intervention proved to be the most effective intervention in Phase 1 and then duplicated the results in Phase 2.

Other songs have been created for disease prevention, but no scientific methods were used to develop them. It is important that a song be developed following a scientific methodology to ensure the song delivers the facts and that the song is appropriate for its intended audience. A song that is easy to remember, repetitive and culturally appropriate will ensure the information is retained and able to be repeated [7]. If a song has too much information or does not hold the target audience’s attention, it will not be listened to, nor get the information desired across. The information could be confusing and do harm by spreading misinformation if the song is too complex [7].

A song named Ebola in Town was released by Liberian musicians Shadow, D-12 and Kuzzy of 2Kings in 2014, a short time after the outbreak of the Ebola disease. The point of the song was to include determinants of the Ebola disease, to educate the public [15]. However, no scientific method was used to ensure the song would be beneficial to spread the information to its target audience in an effective manner. A song came about during an HIV/AIDS epidemic in New York City in the 1990s. The African-American community was at high risk of HIV at the time and the song’s composers wanted to change the education about contracting and preventing HIV. They created a song using a rap video and traditional music for its target population, while using determinants about HIV [16]. However, the researchers did not use any age- and culturally appropriate method to test if the song was effective enough to pass on HIV prevention method messages, and ensure the information was easy to understand and be remembered by its target audience. In addition, the songs contained already known information about the disease. Both the Ebola and the HIV songs were not checked to establish whether they helped with disease prevention, nor is there any mention of how effective the songs were in managing the diseases. A scientific method on how to produce an appropriate song and an appropriate method on how to measure the effectiveness of the song would benefit disease prevention.

Unique results emerged during the present study endorsing the findings of earlier research. Previous studies have shown that information set to music is among the easiest to remember. Music provides a rhythm, a rhyme and often alliteration, all being the key to unlocking information stored in the brain with music acting as a cue. The added melody encourages repetition and thus memorization [17]. When learners at a song-only school expressed how they still remembered the song, it could have been because the song was composed to do so. The song had been approved by experts in their field of music, malaria and education, as well as a focus group of caregivers to be effective due to its repetition, rhythm, and elucidative information to teach young children about malaria [8].

A study done at the Memory Laboratory at Washington University in St Louis, USA investigated why songs from childhood were easier to remember compared to college material learned later. The neuroscientists involved in the study concluded that information set to rhythm and repetition helps with memory retention [17]. Moreover, the Penn State researchers found that people have a ‘cement memory’. This cement memory can only store so much information from studying. They found that because of this cement memory the older you get, the harder it is to remember or learn. This is because cement memory can store only so much information. However, songs and lyrics seem to have no effect on cement memory [18]. These findings are important to address when looking at how learners in the song-only school managed to learn without any lesson plans, and how they managed to learn the material in the song alone.

In addition, a study on using song and music to see if it motivated young children to learn English, used pre- and post-intervention questionnaires to see how well the children were motivated and how well they learned English with their songs. The study found that after four rounds of learning the song, the children began to learn it themselves and would continue practicing it outside the classroom. The English learning study suggested that if music is culturally and age-appropriate, it helps the learner identify more with the song and motivates them to learn it [19]. The song to educate about malaria used in this study was translated into Tshivenda, the local language, and was approved to be age- and culturally appropriate to begin with. These two aspects are complementary and helped the learners in the song-only schools to learn behavioural changes from the song. Learning through this song in the study can help a learner socially as it establishes social bonding between the children that improved their behaviour. The concept of emotional involvement is a way of learning and improving memory retention. Rhyme and repetition help children remember a song better [17]. When emotions and social ties are added to the number of times a song is used, it can increase outside the classroom with other children and even at home.

The parents discussed hearing the song at home and how the learner would sing to them. This helped the parents to learn the song without any interventions that was provided for them. The parents scored two statistical significances in malaria prevention and malaria symptoms in the song-only intervention. The link with the songs being sung at home was confirmed with one of the teachers saying how the parents had heard it at home. The same way the children in song-only schools were able to learn, remember and have retention of a song is then seen in the parents. The parents did score high percentages of malaria knowledge in the pre-intervention questionnaire. However, they did seem to have learned exponentially in the post-intervention questionnaire with regards to malaria prevention methods, and symptoms of malaria. In a study done with dementia patients, the researchers looked at the ‘ripple effect’ music therapy could have on helping dementia patients’ well-being. Musical communities were set up for dementia patients to help them learn and remember better with their musical therapist [4]. The study looked at how well not only dementia patients benefitted from music, but also caregivers, staff and other residents. Findings showed that many people who were outside the dementia community were able to benefit from music that communities had set up, by learning and remembering better. This helped the well-being of everyone involved, not just the dementia patients the music was originally intended for [4]. This ripple effect was experienced with the malaria song where teachers, parents and other learners in different classes or grades also benefitted from the song.

During the study, it was noted that some control groups exhibited higher increases in their scores in certain areas. There were two questions that received statistical significance for the control group. Even though the learners managed to score higher percentages, the following questions saw the learners not actually learning when asked to give examples. It was easy to say “Yes, I know how to prevent malaria”, but then, not provide an accurate prevention method, thus showing that learners did not actually learn the material, yet participants from some of the schools did seem to have learned the material. There was a study that looked at middle schoolchildren learning physics. The results showed that curiosity was a reason why some children sought out answers on their own, thus participants from those control groups managed to learn. The same reason mentioned curiosity and learning the material is thought to have occurred here in this study as well [20]. There were some increases in knowledge growth of the control groups, but not enough to show any real significance.

The proof of concept was agreed to be statistically significant in this study, and it can thus be concluded that the song designed to teach malaria prevention to young children in this study, can help in disease prevention on a broader scale. The song proved to be the most significant out of other interventions and confirmed for the second time that there are promising indications that information transferred to young learners holds promising potential to affect their behaviour for the rest of their lives. This study has manifested the possibility of conveying risk mitigation information to learners with the help of repetitive verses of an age- and culturally appropriate song. Therefore, if other regions, countries or organisations would like to consider this methodology as a possible tool to educate and aid in malaria transmission prevention in young children, the research and protocol could follow the design used in this study (see Fig. 7). Following the same methodology, a song could readily be adapted to reach different target audiences (i.e., different age groups) or convey alternative messages to different audiences to align with the existing malaria status in a region or country (i.e.,when a country moves from malaria control to elimination status).

A few things to consider when planning to develop and implement a temporary or permanent malaria-focused intervention in schools to contribute to knowledge growth and effect behavioural change in a community, region or country: (i) the appropriate authorities need to be approached for approval for such an intervention to be introduced, including the local tribal authority, national malaria control programme, and the governing body and/or principal of the school(s) being targeted; and, (ii) to ensure the intervention does not disrupt a school’s normal activities or class schedule, the relevant teacher(s) that will implement the intervention should be involved during the planning and development phase. Teachers should be reminded of their important role in implementing such interventions in schools and how successful interventions can contribute towards malaria elimination. These two points will aid a local community to accept the intervention, ensure proper implementation and encourage scale-up of the intervention if needed.

## Conclusion

Previous research about the methodology of measuring the success of knowledge growth in disease prevention through a song or other creative intervention could not be identified prior to this study. This research study indicated the song as the most significant and most effective learning intervention in Phase 1 and was confirmed in Phase 2. The age- and culturally appropriate song could be effective to influence positive learning behaviour about malaria prevention. If it succeeds in its message, the song can achieve an important objective to empower children to change behaviour in order to protect against deadly disease. The behavioural changes in children induced by the song are likely to reduce the transmission of malaria by mosquitoes. If the song enables young children to tell or teach their parents or caregivers about malaria, it helps to reduce malaria morbidity and mortality by causing a ripple effect. The song can ultimately manifest those behavioural changes to impact on family and friends of children, allowing for lifestyle changes that could benefit vulnerable populations in malaria areas. This empirically developed song could become an effective tool to help reduce malaria morbidity and mortality not just in the Vhembe District of Limpopo Province in South Africa, but if adapted accordingly through scientific methods to be age- and culturally appropriate, could be used in other high-burden malaria countries worldwide.

## Data Availability

The datasets used and analysed during the study are available from the corresponding author with a reasonable request.
